# Decoding the chemical composition and pharmacological mechanisms of Jiedu Tongluo Tiaogan Formula using high-performance liquid chromatography coupled with network pharmacology-based investigation

**DOI:** 10.18632/aging.203679

**Published:** 2021-11-05

**Authors:** Qi Zhang, Chunli Piao, Wenqi Jin, De Jin, Han Wang, Cheng Tang, Xiaohua Zhao, Naiwen Zhang, Shengnan Gao, Fengmei Lian

**Affiliations:** 1 Changchun University of Chinese Medicine, Changchun 130021, Jilin Province, P.R. China; 2 Shenzhen Hospital, Guangzhou University of Chinese Medicine (Futian), Shenzhen 518000, Guangdong Province, P.R. China; 3 Guang’anmen Hospital, China Academy of Chinese Medical Science, Beijing 100700, P.R. China

**Keywords:** Jiedu Tongluo Tiaogan Formula, type 2 diabetes mellitus, network pharmacology, PI3K/Akt signaling pathway, traditional Chinese medicine

## Abstract

Type 2 diabetes mellitus (T2DM), a chronic low-grade inflammatory disease with high morbidity and mortality, is a serious threat to public health. Previously we demonstrated that a traditional Chinese medicine formulation, Jiedu Tongluo Tiaogan Formula (JDTL), exerted a favorable hypoglycemic effect due to unknown molecular mechanisms involving interactions among JDTL compounds and various cellular components. This study aimed to explore JDTL mechanisms for alleviating hyperglycemia using an integrated strategy incorporating system pharmacology, bioinformatics analysis, and experimental verification. This strategy entailed initial elucidation of JDTL chemical composition using fingerprint analysis via high performance liquid chromatography (HPLC). Next, functions of putative shared target genes and associated pathways were deduced using GO and KEGG pathway enrichment and molecular docking analyses. Ultimately, targets associated with JTDL anti-T2DM effects were found to be functionally associated with biological functions related to lipopolysaccharide and cytokine receptor binding. These results implicated PI3K-Akt signaling pathway involvement in JDTL anti-T2DM effects, as this pathway had been previously shown to play significant roles in glucose and lipid metabolism-related diseases. Furthermore, addition of JDTL to INS-1 and HepG2 cell cultures stimulated cellular mRNA-level and protein-level expression leading to enhanced production of IRS1, Akt, and PI3K. In summary, here JDTL bioactive ingredients, potential targets, and molecular mechanisms underlying JDTL anti-T2DM effects were identified using a multi-component, multi-target, and multi-channel analytical approach, thus providing an important scientific foundation to facilitate development of new drugs mechanistic strategies for preventing and treating T2DM.

## INTRODUCTION

The prevalence of T2DM, one of the greatest emerging health crises worldwide, is projected to increase by >50% globally by 2045 [1], with high T2DM incidence and mortality rates underscoring the huge potential threat of the disease to public health [2]. Generally, poorly controlled diabetes can trigger development of serious complications, including heart attacks, kidney failure, blindness, and nerve damage [3, 4]. However, the current body of accumulated T2DM research cannot fully clarify pathogenic mechanisms associated with the disease, although it has enhanced our understanding of risk factors associated with T2DM pathogenic processes, such as family history, obesity, poor diet, and lack of exercise [5]. Currently T2DM is mainly managed by administration of hypoglycemic medications and insulin. Nevertheless, randomized clinical trials of large numbers of T2DM patients have demonstrated that diabetes medications alone cannot adequately achieve blood glucose control for a large proportion of patients [6]. Therefore, alternative treatments for T2DM are urgently needed.

Traditional Chinese medicine (TCM) treats diseases based on a holistic viewpoint that recognizes the interconnectedness of all body systems. Therefore, TCM possesses advantages based on its pleiotropic, multi-target, prospective, and stable features that enable it to alleviate T2DM and other complex chronic diseases [7]. Moreover, TCM exerts effects that can synergistically lower blood sugar, improve symptoms, improve mood, improve metabolism, improve quality of life, and protect target organs. Thus, TCM when used as an important supplement and alternative medicine can benefit T2DM patients and thus has already gained wide acceptance worldwide when used for this purpose [8]. One such TCM agent, Jiedu Tongluo Tiaogan Formula (JDTL), was formulated based on TCM theory. JDTL consists of five herbal medicines, namely Coptis chinensis Franch (Huanglian), Radix Rhei Et Rhizome (Dahuang), Astragalus propinquus Schischkin (Huangqi), Salvia miltiorrhiza Bunge (Danshen) and Bupleuri Radix (Chaihu), which are combined in relative proportions by weight of 15:9:15:15:10, respectively. In a previous clinical study, administration of JDTL to obese T2DM patients was well-tolerated, effectively reduced blood glucose and glycosylated hemoglobin levels and improved insulin secretion [9]. Meanwhile, results of another study using a rat model of type 2 diabetes suggested that JDTL could alleviate insulin resistance (IR) and reduce apoptosis, while further increasing glucose and lipid metabolism [10]. In addition, other studies have shown that JDTL may inhibit production of inflammation-inducing factors, promote secretion of adiponectin, reduce the adipocyte inflammatory response, and relieve endoplasmic reticulum stress in a multi-target-based manner [11]. Although recent pharmacological studies have indicated that JDTL might exert protective effect on hypoglycemic. However, the pharmacodynamic actual base and atomic biological apparatus of JDTL for treating T2DM is still not clear.

Network pharmacology, an indispensable method for exploring the potential mechanism underlying TCM effects, provides a new research paradigm that may transform the clinical viewpoint of TCM from that of an empirical medicine into that of an evidence-based medicine [12]. Network pharmacology is based on a combination of systems biology and multi-directional pharmacology that constructs multiple networks to explain relationships among drugs, molecules, targets, diseases, and pathways. Using this method, molecular mechanisms underlying TCM prescription effects on T2DM can be explained based on a comprehensive perspective that aligns with the holistic view of TCM as a treatment of disease [13]. For example, a network pharmacology method has been used to define active ingredients and potential targets of Panax notoginseng that participate in alleviation of diabetic retinopathy [14].

**Figure 1:**
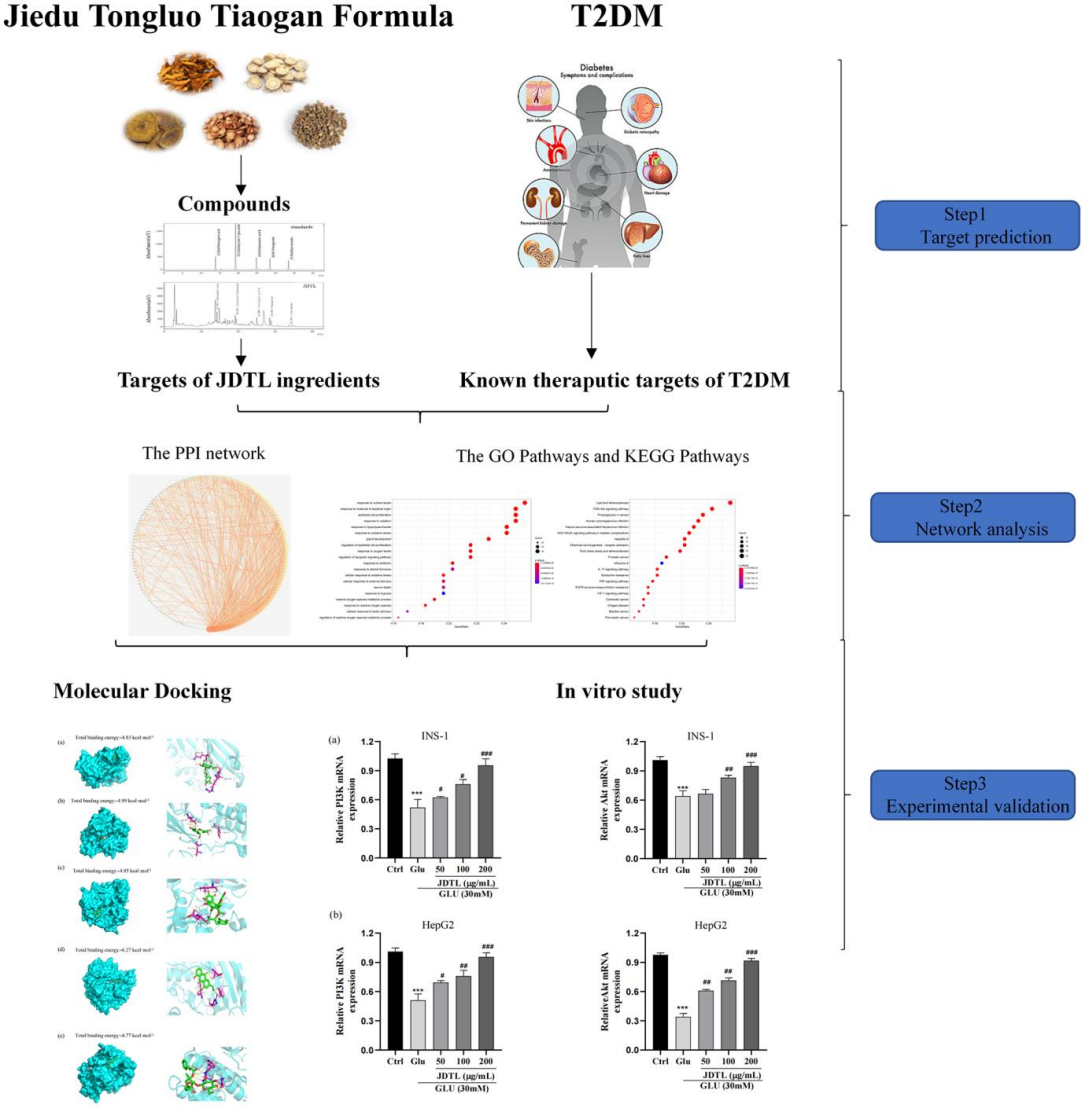
Workflow for the mechanism of JDTL in treating T2DM.

The aim of this study was to investigate potential targets and mechanisms of action associated with JDTL alleviation of T2DM. First, active JDTL ingredients against T2DM were identified. Next, potential molecular mechanisms associated with JDTL effects were determined using network pharmacology-based analysis followed by molecular docking analysis to predict binding affinities among JDTL constituent compounds and putative molecular network hub protein targets. Finally, in order to elucidate mechanisms underlying JDTL alleviation of T2DM, *in vitro* experiments were conducted with INS-1 and HepG2 cells to further explore whether JDTL-associated anti-T2DM mechanisms predicted using integrated network pharmacological analysis played significant roles in observed JDTL anti-T2DM effects in cultured cells. The workflow of this study is shown in Figure 1.

## RESULTS

### Chemical profiling in the water extract of JDTL identified by HPLC

**Figure 2:**
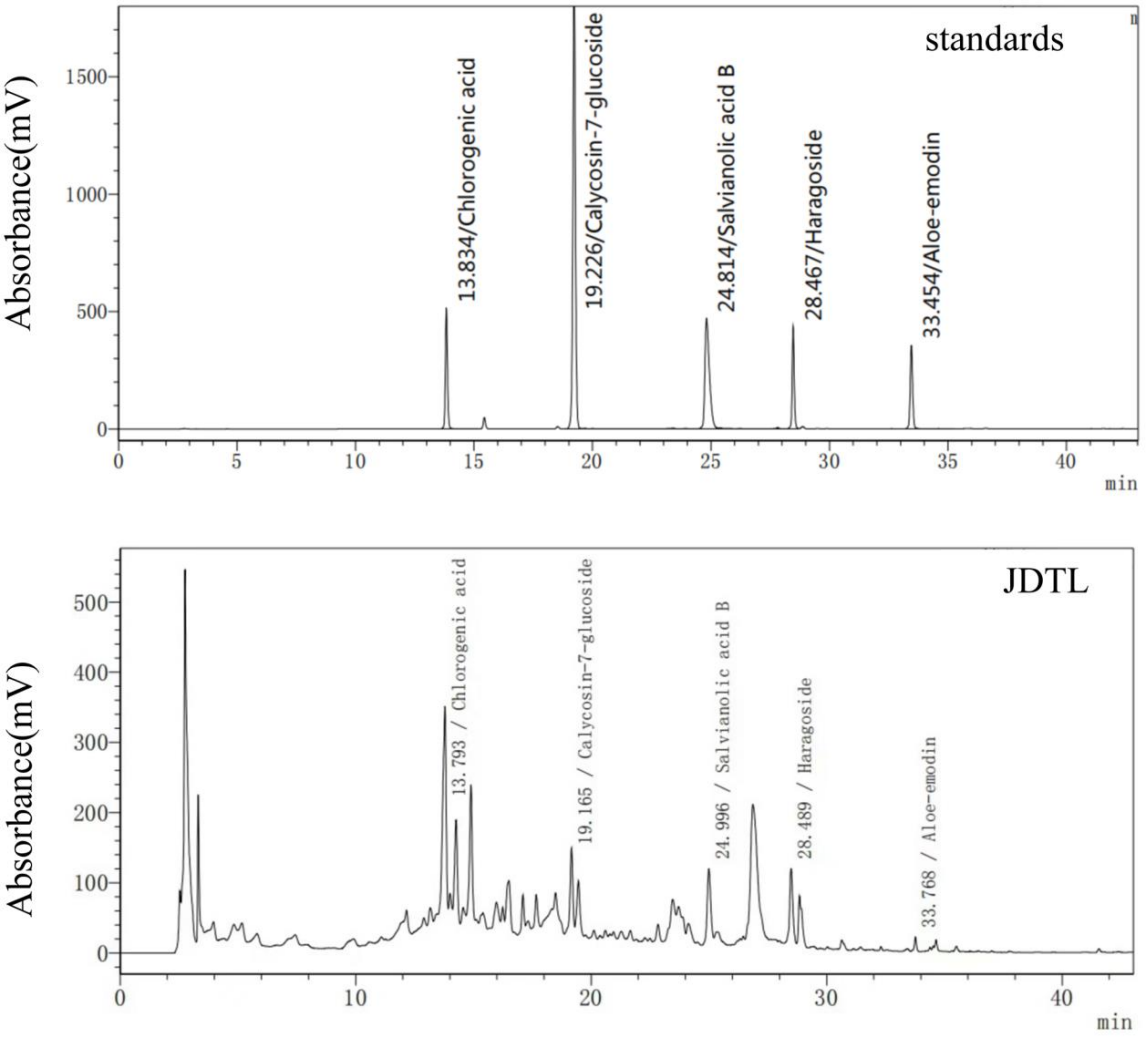
JDTL levels were determined using HPLC.

Based on reference standard data, chromatographic elution behaviors, chemical composition data, mass fragment patterns, and HPLC results, five major peaks corresponding to five major compound constituents of a water-based JDTL extract (Figure 2) were detected. Chemical compounds corresponding to these peaks were then identified, based on HPLC-based retention times of known standards with good reproducibility, as chlorogenic acid, calycosin-7-glucoside, salvianolic acid B, aloe-emodin, and haragoside.

### Target fishing

**Figure 3:**
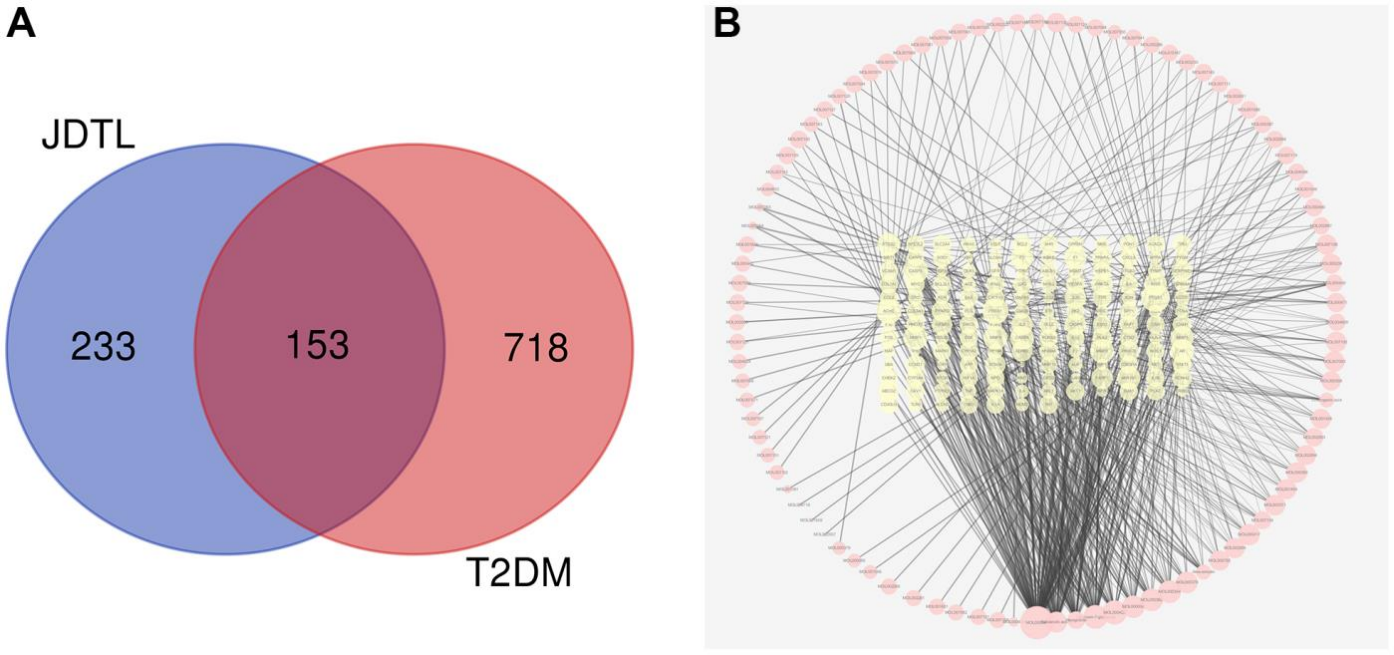
**Common targets and common targets-active ingredients network.** (**A**) Venn diagram of common targets. (**B**) Common targets-active ingredients network. Brick red-colored nodes represent common targets of T2DM and JDTL; yellow nodes represent active ingredients related to common targets.

Following the chemical profiling detected by HPLC system and collected from the existing database, we predicted the putative targets of the chemical constituents containing in JDTL based on the similarities in drug structures and functions as described in our previous studies. A total of 205 active compounds with structural and functional similarities to JDTL constituent compounds were identified by searching the database (Supplementary Table 1). Thereafter, a total of 871 T2DM-related target genes were identified by searching OMIM and GeneCards databases followed by analysis of drug targets and disease targets using Venny 2.1. After eliminating redundant results, we found that JDTL and T2DM shared 153 common targets, as shown in Figure 3A, with the common targets-active ingredients network shown in Figure 3B.

### Protein-protein interaction (PPI) network analyses

**Figure 4:**
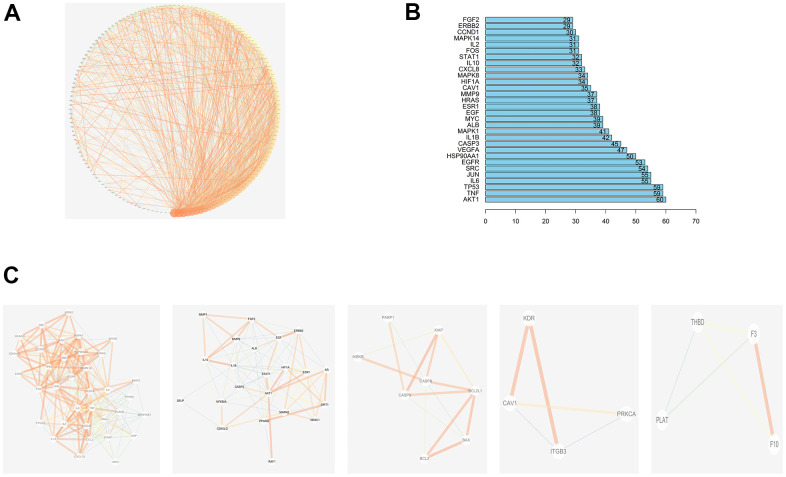
**PPI network of compound-T2DM-related protein.** (**A**)The PPI network by established in the Cytoscape 3.7.2; (**B**)The bar plot of the PPI network; (**C**) Top 5 clustering graphs from the PPI network of T2DM targets.

Shared JDTL and T2DM common targets were imported into the STRING database then their interrelationships were visualized as a PPI network with an average node degree value of 17.1 that contained 148 nodes and 1305 edges. In order to better explain interrelationships among targets, data in tab-separated value (TSV) format were imported into Cytoscape 3.6.2 then results were depicted as network nodes of various sizes and colors based on numbers of combined targets (degree values) for each protein, as shown in Figure 4A.

The degree values of the first 30 genes are shown in Figure 4B, which includes RAC-alpha serine/threonine-protein kinase (AKT1), Tumor necrosis factor (TNF), Tumor Protein P53 (TP53), Interleukin-6 (IL-6), Transcription Factor AP-1(JUN), Sarcoma (SRC), Epidermal growth factor receptor (EGFR), Heat Shock Protein 90 Alpha Family Class A Member 1 (HSP90AA1), Vascular endothelial growth factor A (VEGFA), and Caspase 3 (CASP3) etc. The MCODE plug-in of Cytoscape was then used to generate PPI network clusters of T2DM targets, which are shown in Figure 4C.

### Analyses of enrichment of the GO pathways and KEGG pathways

**Figure 5:**
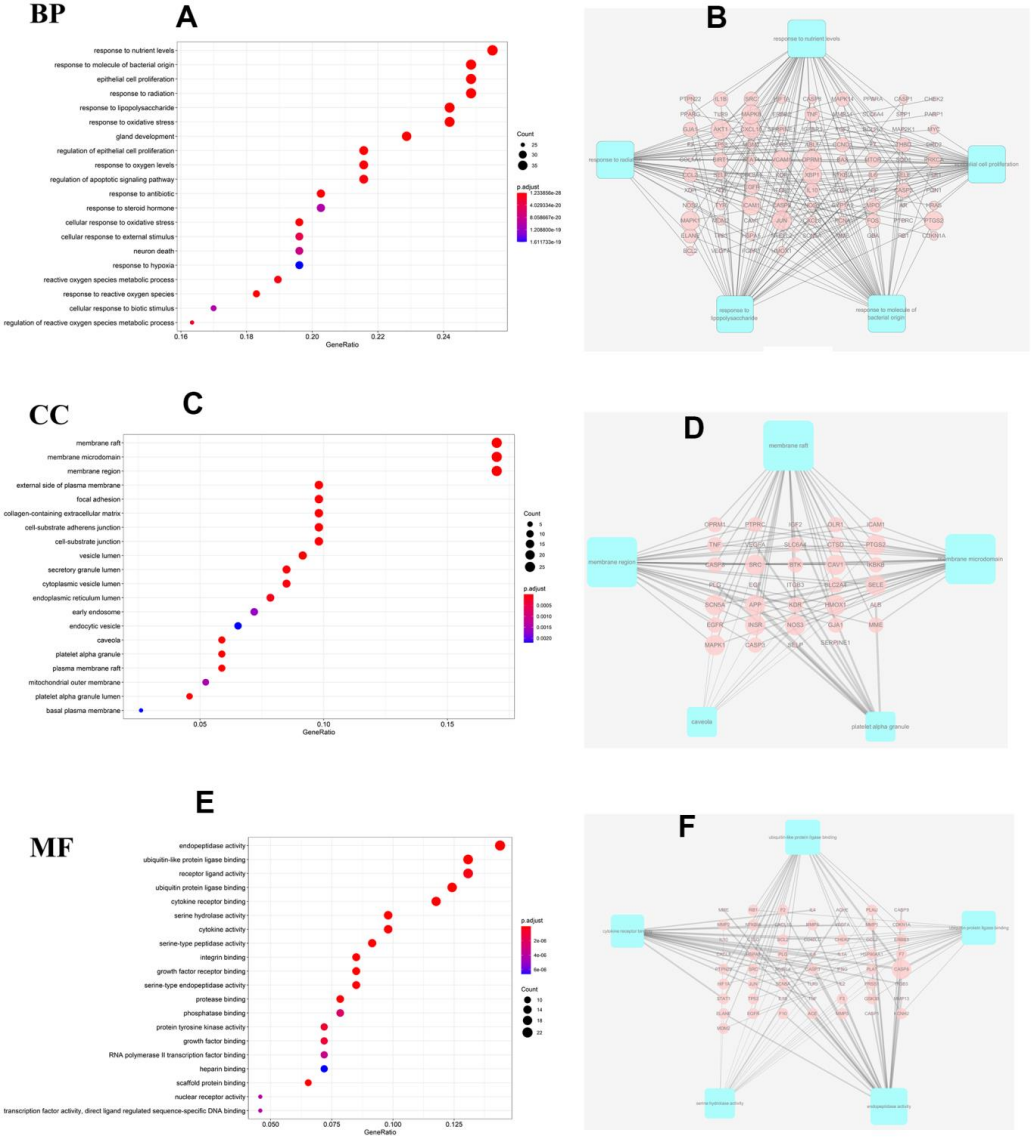
**GO analysis for the major targets of JDTL.** (**A**) Bar chart of biological process categories; (**B**) Sub-network showing the top 5 BP terms and related genes. (**C**) Bar chart of cellular composition categories; (**D**) Sub-network showing the top 5 CC terms and related genes; (**E**) Bar chart of molecular function categories; (**F**) Sub-network showing the top 5 MF terms and related genes (*P*<0.05).

**Figure 6:**
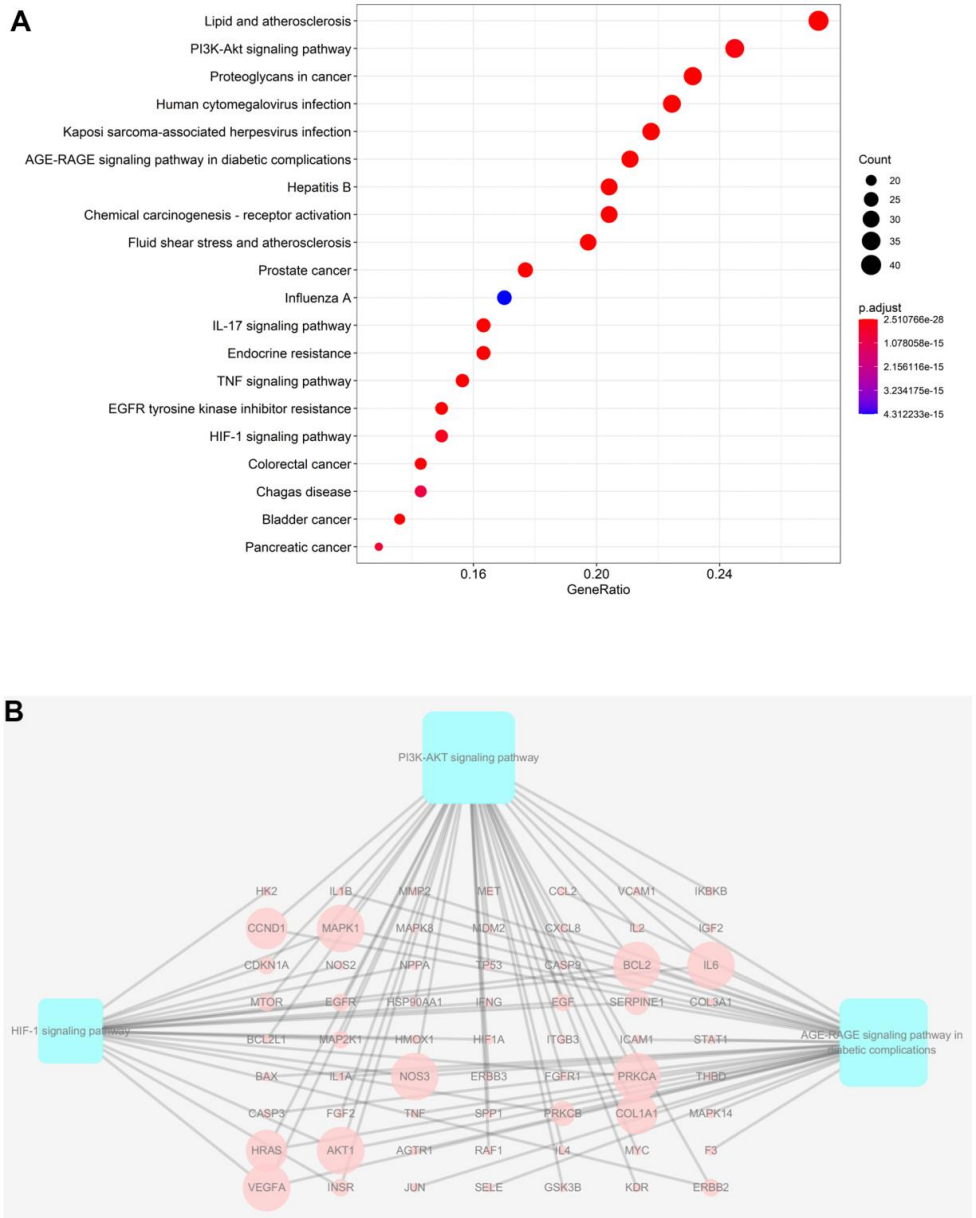
**KEGG analysis of major targets of JDTL.** (**A**) The 20 pathways with the lowest adjusted *P* values. The x-axis and the y-axis represent the counts of the target symbols in each pathway and the main pathway, respectively. (*P* < 0.05) (**B**) Sub-network showing KEGG pathways and related genes.

Taking into account the complex mechanisms underlying JDTL treatment effects on T2DM, integration of key functional terms and pathways were identified via corresponding GO and KEGG database searches using R software that resulted in construction of a complete T2DM-related path diagram. GO analysis was then conducted to assign identified drug targets to the three functional GO categories, namely molecular function (MF), cellular composition (CC), and biological process (BP), as shown in Figure 5A–5F. Notably, the T2DM-related pathways were involved in several biological functions, such as lipopolysaccharide, oxidative stress, cytokine receptor binding, and cell proliferation. Based on KEGG pathway analysis results, 63 targets were significantly associated with the top 20 KEGG pathways (P-values < 0.05), including the PI3K-Akt signaling pathway, AGE-RAGE signaling pathway in diabetic complications, HIF-1 signaling pathway, and others (Figure 6A, 6B). Intriguingly, these pathways had been previously reported to be closely linked to pathogenic mechanisms underlying T2DM and dysfunctional glycolipid metabolism. [8, 15, 16] Thus, JDTL alleviation of T2DM may reflect activities exerted by its constituent compounds that regulate these biological functions.

### Docking analysis results

**Figure 7:**
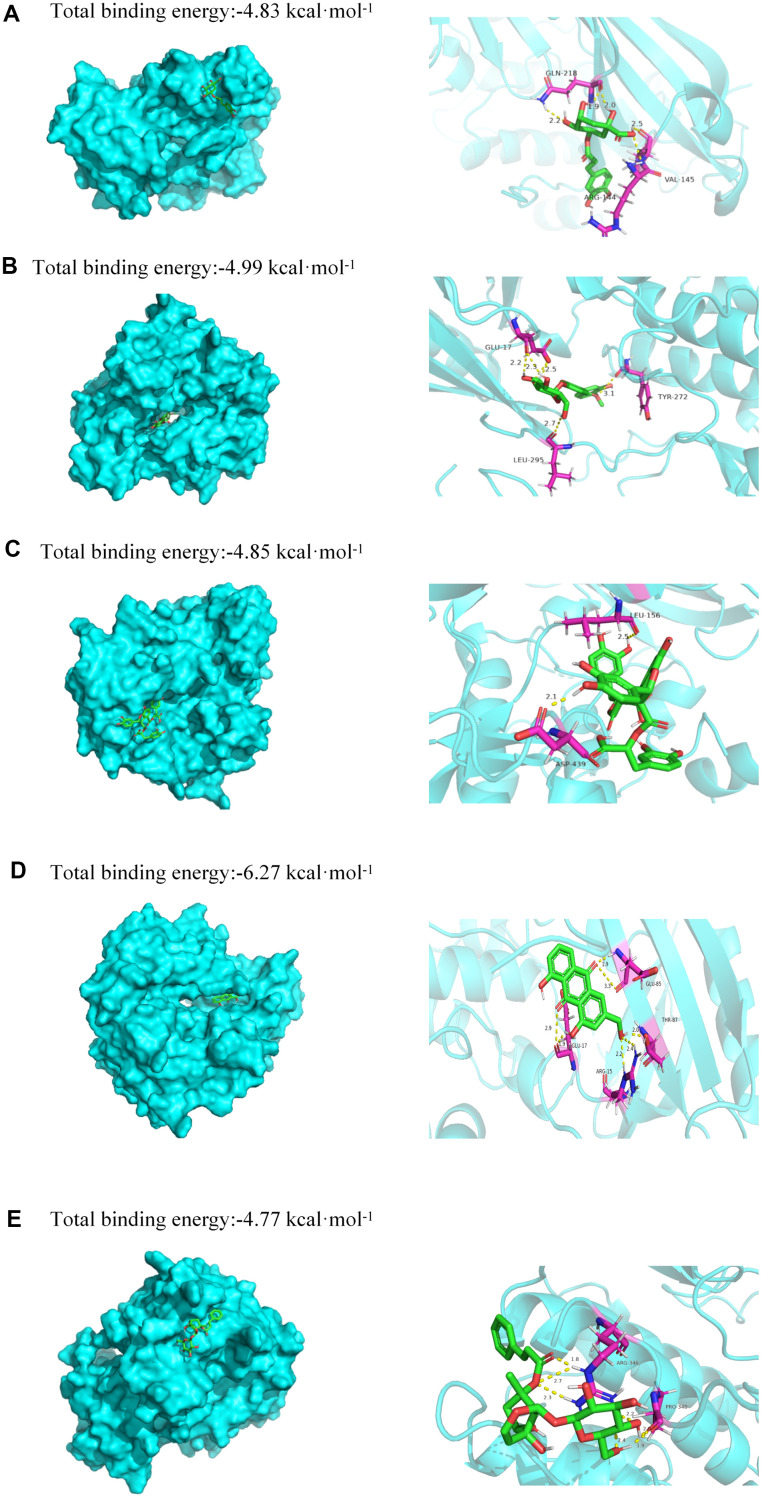
**Molecular models of binding of selected compounds to target proteins.** (**A**) Docking mode and interactions between chlorogenic acid and Akt, (**B**) calycosin-7-glucoside and Akt, (**C**) salvianolic acid B and Akt, (**D**) aloe-emodin and Akt, (**E**) haragoside and Akt.

In this study, molecular docking was used to identify potential binding interactions between JDTL bioactive components and T2DM-associated hub gene targets [17]. To confirm interactions between JDTL components and target cellular proteins, we selected five JDTL components identified by HPLC (chlorogenic acid, calycosin-7-glucoside, salvianolic acid B, haragoside, aloe-emodin), as well as representative target proteins within the three pathways identified using KEGG analysis (Akt, RAGE, HIF1). Next, in order to determine affinity constants for JDTL component binding to putative cellular protein targets, surface plasmon resonance spectroscopy (SPR)-based docking assays were conducted. In this docking assay, three human receptors were retrieved from PDB: Akt (PDB ID:6S9X,2.6 Å), RAGE (PDB ID:3o3u,1.497 Å), HIF1 (PDB ID:4nq0,2.1 Å). The binding energy between the bioactive components and hub proteins is shown in Table 1. In order to verify with a high degree of certainty the reliability of KEGG predictions that Akt acted as a receptor for one or more JDTL constituent compounds, Akt was docked with chlorogenic acid, calycosin-7-glucoside, salvianolic acid B, haragoside, and aloe-emodin. Next, a diagram depicting these putative binding interactions was generated using PyMOL software (Figure 7). From the figure it can be seen that all five major JDTL compounds could form hydrogen bonds with amino acids of Akt as follows: chlorogenic acid with Akt residues GLN-218, ARG-144, and VAL-145; calycosin-7-glucoside with Akt residues TYR-272, GLU-17, and LEU-295; salvianolic acid B with Akt residues ASP-439 and LEU-156; haragoside with Akt residues LYS-297 and LEU-295; aloe-emodin with Akt residues GLU-85, GLU-17, ARG-15, and THR-87. Specifically, free energy (kcal/mol) values for binding interactions between Akt, a PI3K-Akt signaling pathway target protein, and chlorogenic acid, calycosin-7-glucoside, salvianolic acid B, haragoside, and aloe-emodin were -4.83, -4.99, -4.85, -4.77, and -6.27, respectively. These results indicated that high affinity binding interactions between bioactive JDTL components and PI3K-Akt pathway target proteins are possible as support for the premise that JDTL may regulate protein functions associated with the PI3K-Akt pathway.

**Table 1:** The molecular docking result of compound and hub gene.

**Targets**	**Compounds**				
	**Aloe-emodin**	**Calycosin-7-glucoside**	**Chlorogenic acid**	**Harpagoside**	**Salvianolic acid B**
Akt	-6.27	-4.99	-4.83	-4.77	-4.85
RAGE	-3.96	-2.16	-2.35	-3.17	-3.28
HIF1	-5.01	-3.74	-3.11	-3.44	-3.21

### Effect of JDTL on cell viability

**Figure 8:**
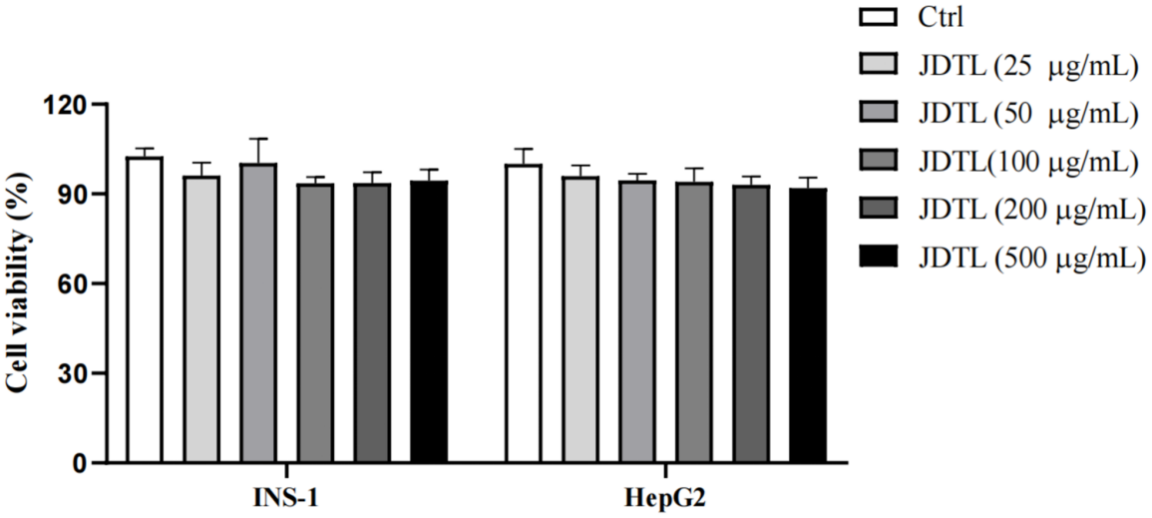
**Effects of JDTL on cell viability.** INS-1 and HepG2 cells were separately treated with different concentrations of JDTL for 24 h then cell viabilities were determined via MTT assay. Values are means ± SD from three independent experiments, ^#^*P* < 0.05 compared to Control.

MTT assays were performed that demonstrated that 24-hour treatment with JDTL at concentrations of 50, 100, and 200 μg/mL had no effect on viability of INS-1 and HepG2 cells (Figure 8). Thus, these JDTL concentrations were used in subsequent experiments.

### Glucose-stimulated insulin secretion (GSIS) and glucose uptake analyses

**Figure 9:**
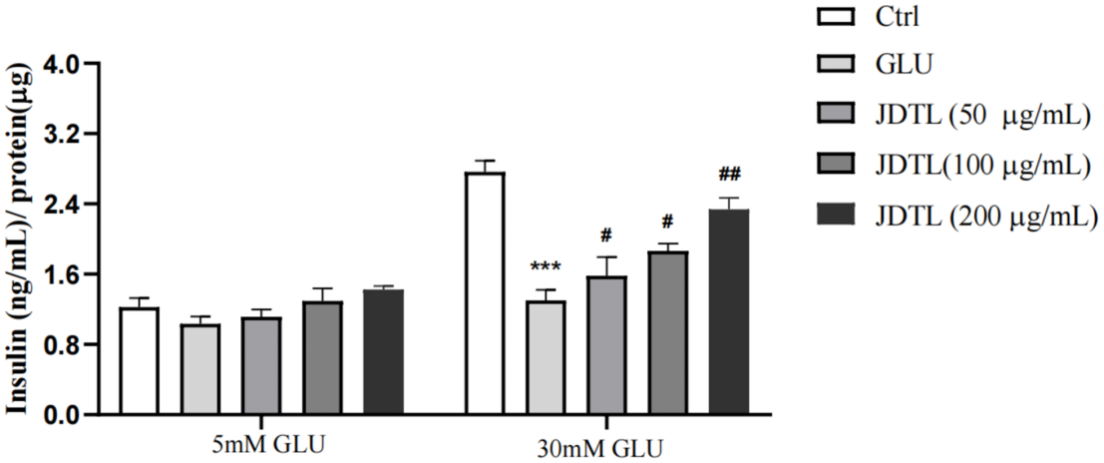
**Effect of JDTL on INS-1 cell insulin secretion.** Values are means ± SD from three independent experiments.

**Figure 10:**
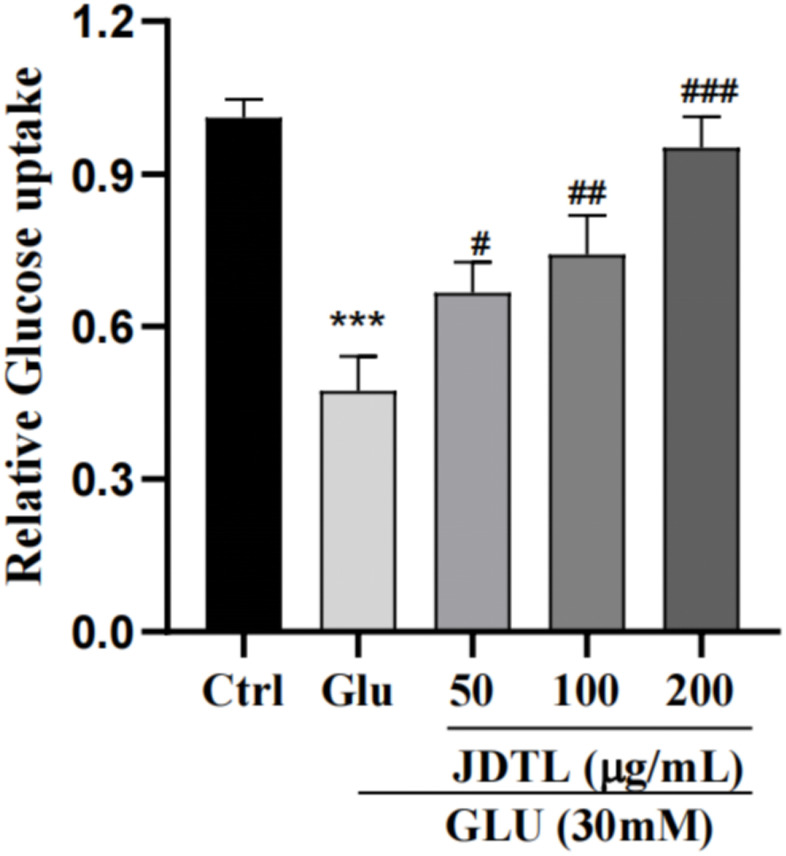
**Effects of JDTL on glucose uptake by HepG2 cells.** Values are means ± SD from three independent experiments.

Results of GSIS analysis showed that after glucose starvation by culture under low glucose (LG) conditions (5.0 mM glucose) for one hour, normal GSIS by INS-1 cells was observed for the 5.0 mM group, while relatively lower levels of GSIS by INS-1 cells in high-glucose (HG) groups were observed. Following the same glucose starvation protocol, INS-1 cells were stimulated with a high level of glucose (30 mM) that led to decreased insulin secretion as compared to that of the control group. Conversely, insulin secretion function was greatly increased after JDTL treatment (Figure 9). In addition, HepG2 cells exposed to 30 mM glucose for 24 h followed by incubation with insulin (100 nM) for 30 min exhibited marked decreased 2-deoxy-2-[(7-nitro-2,1,3-benzoxadiazol-4-yl) amino]-D-glucose (2-NBDG) uptake as compared to that of control cells that was countered by addition of JDTL, resulting in enhanced 2-NBDG uptake by HepG2 cells in all JDTL treatment groups (Figure 10).

### Effect of JDTL on PI3K-Akt signaling pathway target gene expression

According to network pharmacology analysis and molecular docking results obtained in this study, we speculated that JDTL treatment-based alleviation of T2DM might reflect JDTL-induced regulation of glucose and lipid metabolism through activation of the PI3K-Akt signaling pathway. To test this hypothesis, Western blot and qRT-PCR analyses were conducted to determine total protein-level and mRNA-level expression associated with PI3K and Akt target proteins.

#### PI3K and Akt mRNA-level expression in INS-1 and HepG2 cells

**Figure 11:**
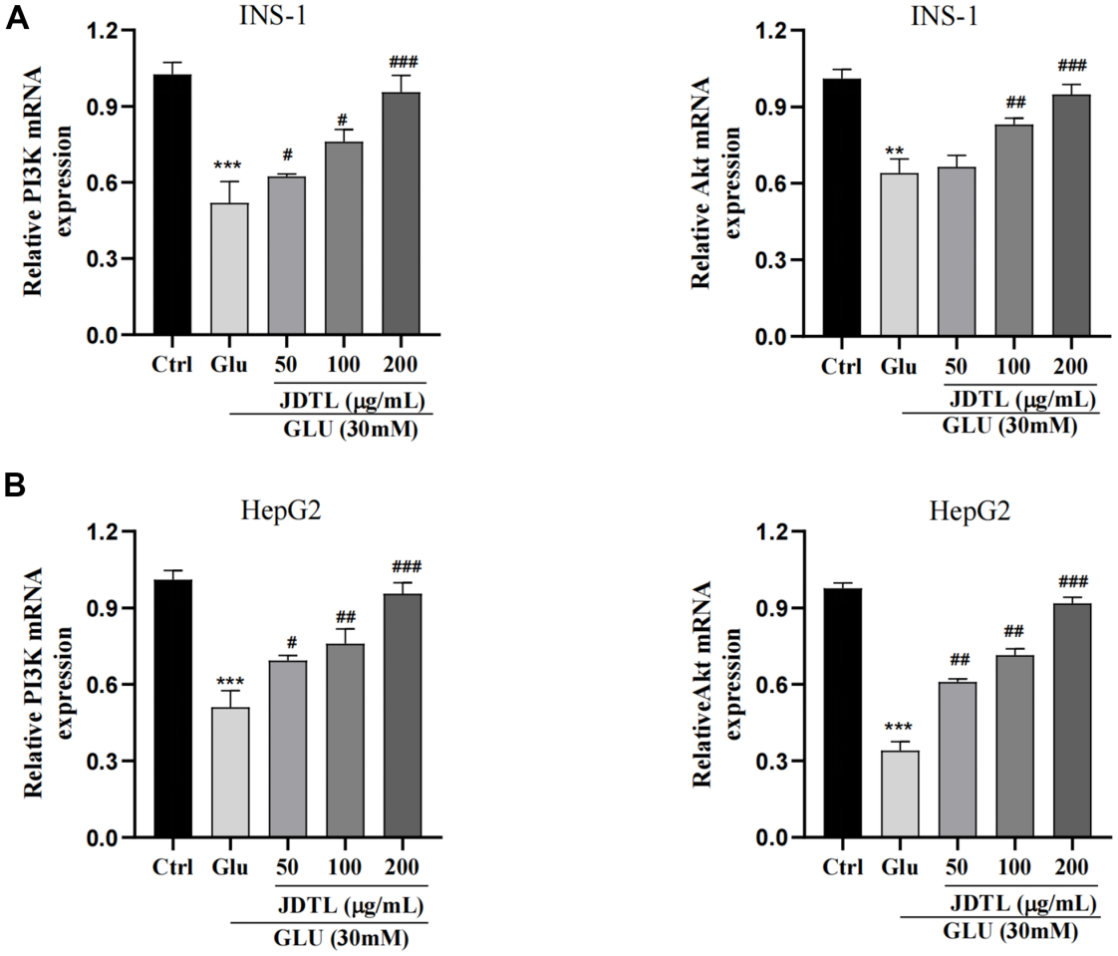
(**A**, **B**) Effects of JDTL on mRNA expression levels associated with PI3K and Akt expression. (**A**) in INS-1 cells. (**B**) in HepG2 cells. Values are expressed as means ± SD from three independent experiments. ***P < 0.01. ^#^*P* < 0.05, ^##^*P* < 0.01, ^###^*P* < 0.001 as compared to the model.

PI3K and Akt mRNA expression levels in INS-1 and HepG2 cells were assessed using qRT-PCR. As shown in Figure 11A, 11B, cell exposure to high glucose levels was associated with significantly decreased mRNA levels associated with PI3K and Akt expression that were both increased after treatment of both cell types with JDTL.

#### The protein expression of PI3K and Akt in INS-1 cells and HepG2 cells

**Figure 12:**
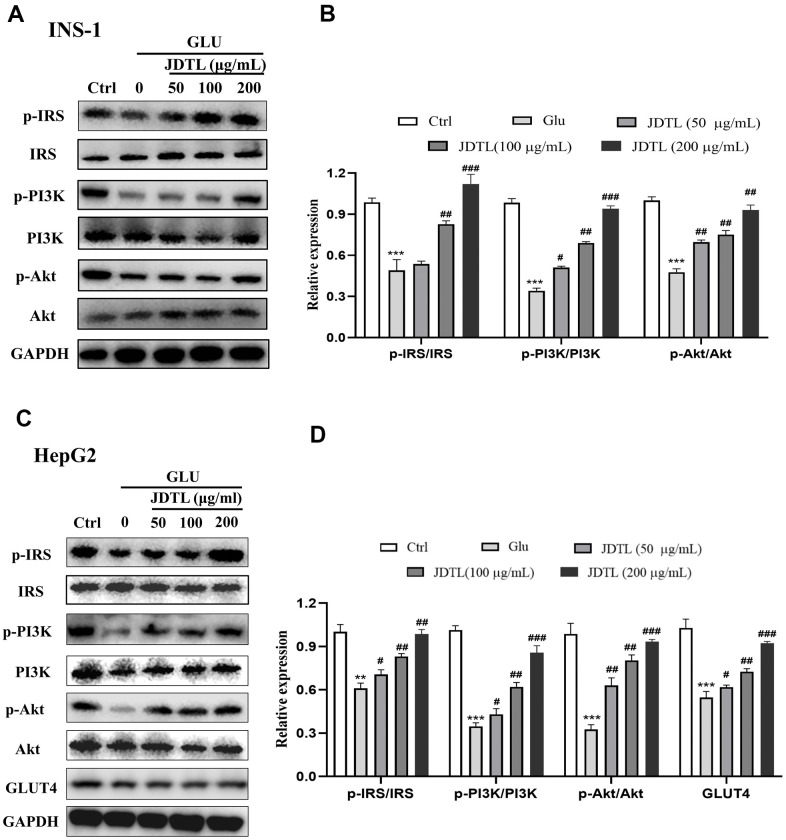
(**A**–**D**) Effects of JDTL on IRS-1/PI3K/Akt protein expression. (**A**) in INS-1 cells. (**C**) in HepG2 cells. Cell lysates were subjected to Western blot analysis using GAPDH as loading control. (**B**, **D**) Quantification of band intensities relative to that of GAPDH bands using Image J software. Values are expressed as means ± SD from three independent experiments. ***P < 0.01. ^#^*P* < 0.05, ^##^*P* < 0.01, ^###^*P* < 0.001 as compared to the model.

**Figure 13:**
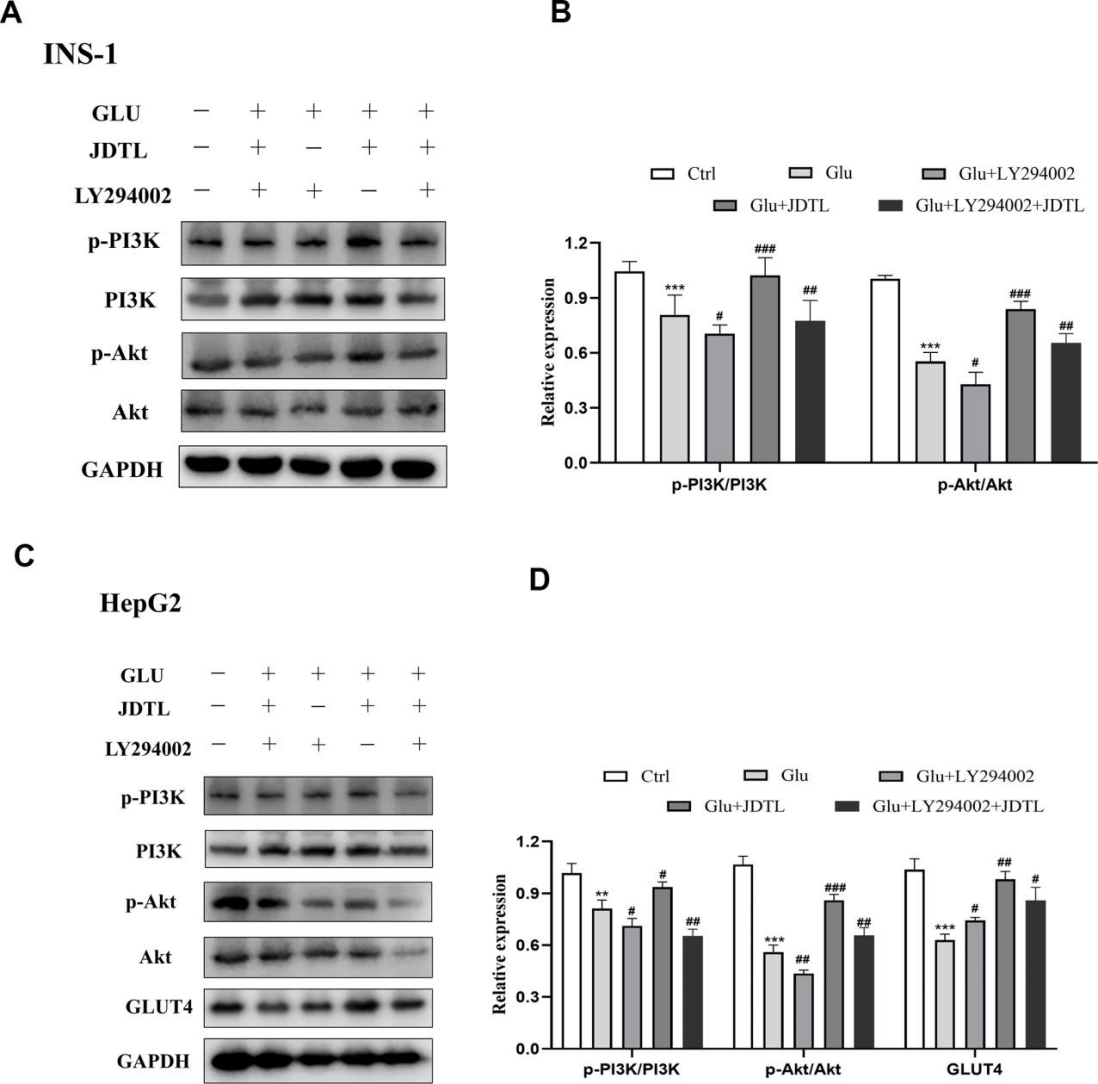
(**A**–**D**) Inhibitory effect of PI3K inhibitor on PI3K-Akt signaling pathway in INS-1 and HepG2 cells. (**A**) Effect of PI3K inhibitor on p-PI3K and p-Akt levels in INS-1 cells. (**C**) Effect of PI3K inhibitor on p-PI3K, p-Akt, and GLUT4 levels in HepG2 cells. Cells were treated with or without 20 μM PI3K inhibitor LY294002 for 24 h. (**B**, **D**) Quantification of bands relative to GAPDH intensities using Image J software. Values are expressed as means ± SD from three independent experiments. ***P < 0.01. ^#^*P* < 0.05, ^##^*P* < 0.01, ^###^*P* < 0.001 as compared to the model.

In order to determine molecular mechanisms underlying JDTL effects, we conducted network pharmacological analysis of key proteins within integrated “T2DM-related pathways,” including the PI3K-Akt signaling pathway. To confirm activation of the PI3K-Akt signaling pathway, we measured IRS, PI3K and Akt proteins in INS-1 cells and HepG2 cells using Western blotting. To assess PI3K-Akt signaling pathway activation status, we measured IRS, PI3K, and Akt protein levels in INS-1 and HepG2 cells using Western blot analysis. The results showed that the HG-exposed INS-1 group cells exhibited significantly decreased expression of p-IRS, p-PI3K, and p-Akt proteins relative to corresponding control levels of these proteins, while JDTL treatment inhibited decreases in levels of these proteins in HG-exposed INS-1 cells (Figure 12A, 12B). GLUT4 is an insulin-regulated transmembrane glucose transporter that controls glucose homeostasis and is a downstream target of the PI3K-Akt pathway [18], whereby decreases in GLUT4 expression reflect impairment of the PI3K-Akt signaling pathway [19]. As shown in Figure 12C, 12D, expression levels of p-IRS, p-PI3K, p-Akt, and GLUT4 proteins were significantly upregulated after JDTL treatment of HepG2 cells as compared to corresponding control cell levels of these proteins. To determine whether JDTL treatment alleviated T2DM by regulating expression of p-PI3K, cells were incubated with or without 20 μM PI3K inhibitor (LY294002) and/or 200 μg/mL of JDTL for 24 h. The results revealed that JDTL treatment significantly upregulated expression of p-PI3K and p-Akt; these results were contrary to results obtained for LY294002 treatment alone (Figure 13A, 13B). Taken together, these data suggest that JDTL treatment activated the PI3K-Akt signaling pathway in INS-1 and HepG2 cells.

## DISCUSSION

T2DM is one of the most prominent and detrimental chronic diseases of humans, due to its many complications, low cure rate, and management challenges. Nevertheless, T2DM may be treatable with TCM formulations, which are based on highly intuitive principles for achieving clinically curative effects that have shown to be effective in clinical practice [20].

Notably, results of various research reports suggest that JDTL active ingredients may regulate glucose levels. For example, bioactive components of Coptidis Rhizoma have been shown to improve disordered glucose and lipid metabolism in T2DM animal models by acting on a variety of signaling pathways to ultimately reduce effects of glucose toxicity [21, 22]. Meanwhile, huangqi polysaccharides have been used to prevent and treat T2DM, due to their abilities to enhance immunity and improve insulin sensitivity [23]. Additionally, Salvia miltiorrhiza Bunge has been shown to regulate colonic motility and intestinal neuronal disturbances by restoring the intestinal mechanical barrier of diabetic mice [24]. However, TCM formulations are often complex, making it difficult for researchers to determine their mechanisms of action for alleviating diseases. Nevertheless, in the field of TCM research, network pharmacology analysis in combination with bioinformatics analysis has been used to identify and analyze assorted biologic targets and interactions associated with diseases so as to comprehensively explain molecular mechanisms of action of some TCM formulations [25, 26].

### Underlying mechanisms of JDTL alleviation of T2DM as revealed using HPLC and network analysis

In this study, we attempted to identify JDTL active components, potential targets, and mechanisms of action using HPLC and network pharmacology. HPLC results revealed that bioactive components of JDTL included chlorogenic acid, calycosin-7-glucoside, salvianolic acid B, aloe-emodin, and haragoside, among others. Network pharmacology analysis revealed that mechanisms underlying JDTL effects for alleviating T2DM involved a total of 210 active components, 153 potential targets, and 167 related signaling pathways. Specifically, network hub proteins (e.g., AKT1, TNF, TP53, IL-6, JUN, SRC, EGFR, HSP90AA1, VEGF, and CASP3) and certain signaling pathways (e.g., PI3K-Akt signaling pathway, HIF-1 signaling pathway, AGE-RAGE signaling pathway in diabetic complications) were found to be associated with mechanisms linked to JDTL anti-T2DM effects. Next, AutoDockTools was used to conduct molecular docking of JDTL compounds with hub proteins belonging to the three key pathways predicted by KEGG analysis to be associated with JDTL anti-T2DM effects. After that, KEGG pathway enrichment and molecular docking analyses focusing on the PI3K-Akt signaling pathway were conducted to more precisely determine the JDTL mechanism of action. Finally, *in vitro* experiments were conducted to assess the reliability of our network pharmacology prediction results. The results of these analyses led to identification of potential JDTL bioactive compounds, targets and signaling pathways associated with JDTL alleviation of T2DM that we report here for the first time. In addition, results of PPI network topology analysis highlighted AKT1, TNF, TP53, IL6, and JUN as the top five most important JDTL targets. Moreover, molecular docking results demonstrated that JDTL constituent compounds chlorogenic acid, calycosin-7-glucoside, salvianolic acid B, aloe-emodin, and haragoside could interact with Akt with good binding affinity.

### Functional analysis of candidate targets of JDTL anti-T2DM effects

Pivotal JDTL active ingredients most highly related to biological functions associated with lipopolysaccharide and cell proliferation included chlorogenic acid, calycosin-7-glucoside, salvianolic acid B, aloe-emodin, and haragoside. These compounds may thus play key roles in JDTL amelioration of T2DM, warranting future studies. Intriguingly, chlorogenic acid has been shown to modulate lipid and glucose metabolic regulation associated with both genetically and environmentally induced metabolic disorders, such as hepatic steatosis, cardiovascular disease, diabetes, and obesity [27, 28]. Calycosin-7-glucoside, the main chemical component of Astragalus membranaceus, exerts numerous biological effects, such as anti-inflammatory, anti-tumor, anti-aging, antioxidant, and vasodilation-related effects [29]. Moreover, it can increase the antioxidant capacity of cells, reduce cellular oxidative stress damage, and alleviate IR [30]. Salvianolic acid B is a natural product purified from Salvia miltiorrhiza (a member of the plant family Labiatae) that possesses known antioxidant, anti-inflammatory, neuroprotective, and other pharmacological activities. It has been reported that salvianolic acid B exerts a protective effect against injury of cells of a pancreatic islet cell line [31, 32]. Quercetin has many biological effects, such as anti-inflammatory, anti-tumor, anti-aging, antioxidant, vasodilator. It is a potential drug to improve T2DM and its cardiovascular complications [33, 34]. Kaempferol has been shown to prevent obesity and IR induced in mice fed a high-fat diet, effects that may be due to amelioration of IR [35]. In addition, kaempferol appears to protect cardiomyocytes from hypoxia reoxygenation-associated damage and endothelial cells from oxidative stress [36]. Meanwhile, isorhamnetin has been shown to alleviate glucolipotoxicity-induced injury of skeletal muscle cells that may be related to inhibition of JAK-STAT pathway activity [37]. Thus, the anti-diabetes effect of JDTL may be an integrated effect of the above components. These results indicate that JDTL has a synergistic effect in the treatment of T2DM, which reflects the characteristics of multi-component collaborative improvement of blood glucose.

Akt is a serine/threonine protein kinase, a downstream signal protein of PI3K, which mediates cell proliferation, migration, differentiation, survival, or glucose metabolism [38, 39]. Akt is a key insulin signaling molecule in the body, which has a significant regulatory effect on glucose and lipid metabolism. Some experimental results showed that Akt was over expressed in the kidney of diabetic rats, and liraglutide could reduce the expression level of Akt1, hinder renal fibrosis, relieve oxidative stress and improve diabetic nephropathy [40]. TNF is one of the key genes of the T2DM pathway [41], is a key proinflammatory adipocytokine that is released from adipocytes and adipose tissue-derived mesenchymal stem cells, especially in subjects afflicted with certain metabolic diseases [42]. IL6, another hub gene in the PPI network, is a pro-inflammatory cytokine that also plays a complex role in T2DM pathogenesis and acts as a cytokine with multiple functions that can directly damage islet β cells and induce IR [43]. Notably, downregulation of IL-6 can effectively alleviate lipopolysaccharide (LPS)-induced T2DM. MAPK1 and MAPK8 are members of the mitogen-activated protein (MAP) kinase family. MAPK1, also known as extracellular signal-regulated kinase (ERK), is mainly involved in cell proliferation and differentiation, oxidative stress, and other physiological functions, with previous studies showing that inhibition of MAPK1 protein expression could improve myocardial fibrosis in diabetic rats and reduce HG-induced myocardial damage [44]. In addition, other studies have shown that both insulin secretion and β cell mass dynamics are regulated by related kinases belonging to the AMPK family [45].

### Regulatory effects of JDTL on T2DM-related cytokines and proteins

**Figure 14:**
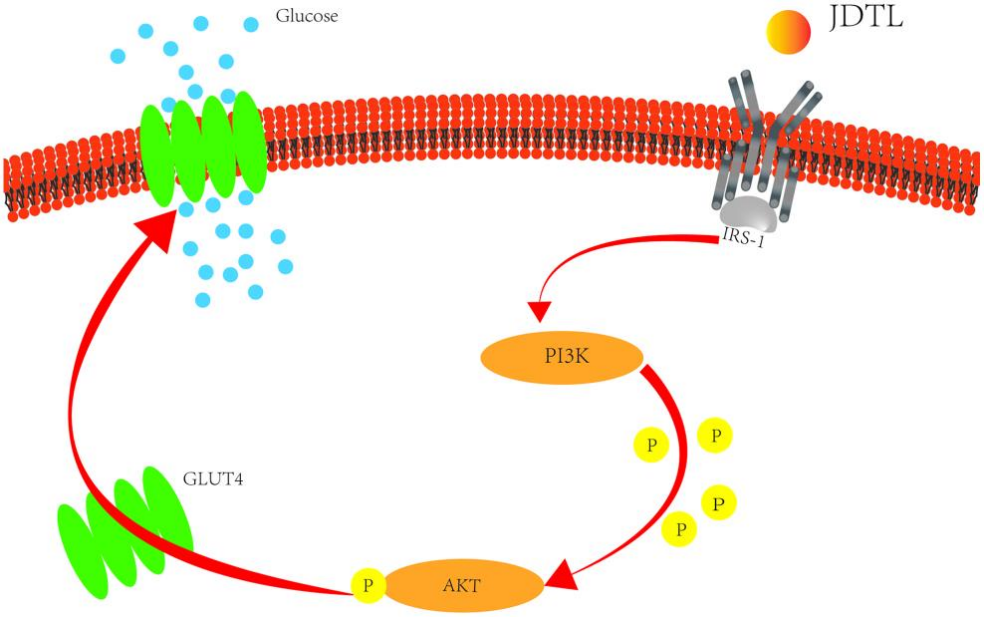
Potential T2DM metabolic pathways regulated by treatment with JDTL.

To determine whether JDTL activated the PI3K-Akt signaling pathway, levels of p-Akt and p-PI3K were assayed, since results of a literature search conducted here indicated that PI3K-Akt pathway core proteins Akt, TNF, and IL6 were closely related to glucometabolic processes, including T2DM. T2DM is a metabolic disease characterized by long-term hyperglycemia caused by potential metabolic dysfunctions, such as IR of muscle and liver tissues and decreased insulin secretion by pancreatic β-cells [46]. Normally β-cells can increase insulin release to maintain normal glucose tolerance. However, when β-cells fail to compensate for decreased insulin sensitivity, T2DM may occur [47]. Therefore, in order to explore the protective mechanism of JDTL on islet cells, we chose to experimentally assess JDTL effects on rat islet cells (INS-1). In T2DM, IR can be defined as a metabolic state in which insulin action (insulin sensitivity) is relatively lower than that occurring under normal physiological conditions. IR occurs in target organs, such as liver, muscle, and adipose tissues [48]. To further verify the hypoglycemic effect of JDTL, a HepG2 cell model of IR was established due to HepG2 cell characteristics of low glucose consumption based on a requirement for high insulin levels that mimic T2DM symptoms. We then used this model to explore molecular mechanisms underlying JDTL effects on INS-1 and HepG2 cell insulin secretion and glucose uptake, respectively. Using network analysis and functional enrichment analysis, we demonstrated that several candidate JDTL targets were associated with the PI3K-Akt signaling pathway, a survival-promoting signaling pathway with key roles in islet cell processes, such as proliferation, growth, apoptosis, and lipid metabolism, that acts by mediating growth factor signaling [49, 50]. It is the main pathway of insulin signal transduction and the main signal pathway of regulating blood glucose. The abnormality of PI3K-Akt signaling pathway is an important cause of diabetes [51]. In fact, studies have found that decreased phosphorylation of PI3K-Akt proteins is the main mechanistic cause of IR [52]. According to the results of *in vitro* cell experiments conducted in this work, JDTL treatment markedly increased levels of PI3K and Akt proteins that exhibited abnormally decreased expression in HG-exposed INS-1 and HepG2 cells, implying that JDTL exerted regulatory effects on the PI3K-Akt signaling pathway. Finally, we added the selective PI3K inhibitor LY294002 to cells to verify that JDTL acted through the PI3K-Akt pathway, as predicted here using network pharmacology and molecular docking analyses. Ultimately, the results of this study demonstrated that JDTL exerted a therapeutic anti-T2DM effect based on a mechanism of action predicted in advance using network pharmacology analysis (Figure 14).

JDTL, a TCM formulation, shares specific features common to all TCM formulations, including multi-component, multi-target, and multi-pathway mechanisms of action. Although several major components of JDTL were identified by HPLC, the direct relationship between active JDTL ingredients and their targets associated with JDTL anti-T2DM remains unknown. In the present study, the PI3K-Akt signaling pathway was predicted to be a target of JDTL action based on results of network pharmacology analysis that were subsequently confirmed experimentally. Nevertheless, other cellular pathways may also be targeted by JDTL, warranting further animal and clinical studies to more comprehensively uncover mechanisms responsible for observed anti-T2DM effects of JDTL.

## CONCLUSIONS

In conclusion, due to the complexity of JDTL, a prescribed TCM formulation, its components are complex and diverse, network pharmacology analysis, molecular docking, and *in vitro* experiments were conducted in this work to explore JDTL bioactive components, targets, and pathways. Ultimately, we predicted that 153 active JDTL ingredients may directly interact with T2DM targets, with 10 top potential targets and 167 related signaling pathways confirmed to have relevance to JDTL beneficial anti-T2DM effects. Moreover, results of cell-based experiments showed that JDTL treatment could increase insulin secretion by INS-1 cells and improve glucose uptake by HepG2 cells by targeting key proteins within the PI3K-Akt signaling pathway and other key pathways with possible relevance to T2DM. Therefore, JDTL exerts good hypoglycemic and insulin secretion-promoting effects that highlight its great potential as an anti-diabetes drug.

## MATERIALS AND METHODS

### Screening to identify JDTL active components

Oral bioavailability (OB) and drug similarity (DL) are two important parameters used to evaluate active components of drugs during research and development stages to assess drug absorption and distribution characteristics in the human body. In this study, active compounds of JDTL were screened against the Traditional Chinese Medicine Systems Pharmacology (TCMSP) chemical database based on thresholds of OB ≥ 30% and DL ≥ 0.18. Next, prospective targets were analyzed using the UniProt knowledge database (https://www.uniprot.org/).

### Identification of gene targets associated with T2DM

To investigate roles of JDTL putative targets in T2DM, we collected a list of known T2DM-related genes from two databases. One database, the GeneCards v4.14 (http://www.genecards.org/) network library, was searched for T2DM-related targets, with results filtered based on a correlation value of ≥30 as the screening parameter. The other database, the Online Mendelian Inheritance in Man (OMIM) database (http://www.omim.org/), was used for target evaluation. In order to ensure the accurateness of the final results, redundant and erroneous targets were removed then the final set of prospective targets was evaluated using UniProt.

### Construction of a protein-protein interaction (PPI) network

Intersecting results obtained for drug targets and T2DM-related genes were depicted using a Venn diagram then intersecting results were imported into the STRING database (https://string-db.org/). Interaction relationship results were limited to the species “Homo sapiens” with confidence scores of >0.7. Next, the results were analyzed using Cytoscape 3.6.2 to construct a PPI network.

### Cluster analysis

Cluster analysis is a type of statistical analysis technology that divides research objects into relatively homogeneous clusters. We used cluster analysis to screen out redundant or similar nodes and protein complexes from the complex PPI network then the MCODE plug-in in Cytoscape (version 3.6.2) was used to perform cluster analysis of PPI networks. Screening conditions included node score cutoff = 0.2, k core = 2, maximum depth = 100, and degree cutoff = 2.

### Enrichment of gene ontology (GO) pathway and the kyoto encyclopedia of genes and genomes (KEGG) pathway

In order to investigate key cell functions and signaling pathways that were mainly affected by key T2DM-associated targets of JDTL treatment, we used clusterProfile version 3.6.2 of the R package of Bioconductor (http://www.bioconductor.org/about/) to perform GO function enrichment analysis on common targets, draw GO analysis entry diagrams, and obtain GO terms from categories of biological process (BP), molecular function (MF) and cell component (CC) followed by selection of results with P values < 0.05. Next, targets enriched for common KEGG pathways (P < 0.05) were identified and the results were expressed as a bubble chart.

### Active ingredients and hub targets interaction analysis

Hub protein targets and pivotal JDTL active ingredients were analyzed as receptors and ligands, respectively, then proteins were dewatered and deliganded using PyMOL 2.3.4 software, core gene targets were hydrogenated and charge calculated using AutoDockTools [53], and the results were saved in the pdbqt format. Upon completion, ligand-receptor molecular docking was performed using AutoDockVina. The stability of the binding of the receptors and ligands depends on the binding energy. The lower the binding energy, the more stable the binding conformation of the receptor and the ligand. The binding energy -4.5 kcal/mol ^-1^ is set as the threshold to determine whether the binding of the receptors and ligands is good or not.

### Preparation of the JDTL

Herbs used to formulate JDTL were provided by the Department of Pharmacy of the Affiliated Hospital of Changchun University of Chinese Medicine (Jilin, China). According to the standard procedure described in the Chinese Pharmacopoeia (2015 edition), 15 g of Huanglian (Coptis chinensis Franch), 9 g of Dahuang (Radix Rhei Et Rhizome), 15 g of Huangqi (Astragalus propinquus Schischkin), 15 g of Danshen (Salvia miltiorrhiza Bunge), and 10 g of Chaihu (Bupleuri Radix) were suspended in 1000 mL of reverse osmosis water then the mixture was simmered at 100° C for approximately 30 min until the volume was 300 mL to generate an extract. This procedure was repeated three times to generate a final aqueous extract, which was filtered and centrifuged then the supernatant was freeze-dried under vacuum to produce a powder with a yield of 13.64% [54]. The powder was dissolved in deionized water to form a concentrated solution (100 mg/mL) that was then filtered (0.2 μm), sterilized, then diluted for biological studies. In order to ensure that the JDTL formulation was of high quality, we analyzed its composition using high-performance liquid chromatography (HPLC). This formulation was used in *in vitro* experiments conducted using INS-1 and HepG2 cells to validate network analysis results.

### Cells and cell cultures

INS-1 and HepG2 cells were purchased from the Cell Center of Peking Union Medical College Hospital and maintained separately in RPMI-1640 medium (Gibco, USA) or DMEM (Gibco or Thermo Fisher Scientific) containing 10% fetal bovine serum, 100 U/mL penicillin, and 100 μg/mL streptomycin in an incubator maintained at 37° C with 5% CO2 and humidification. Throughout the entire cell culture process, strict sterility was maintained. In order to explore JDTL effects and its mechanism of action, INS-1 and HepG2 cells were incubated with 30 mM glucose (HG) for 24 h in the presence or absence of JDTL (50-200 mg/mL). Meanwhile, cells of the control group were cultured in 1640 or DMEM medium for 24 h. All cells used in experiments were initially collected from maintenance cultures in logarithmic growth phase.

### Cell viability assay

INS-1 and HepG2 cells were respectively seeded into wells of 96-well plates (10^4^ cells/well) then were cultured for 24 h prior to supplementation with JDTL of known concentration (0, 50, 100, 200, or 500 mmol/L). Next, spent medium was removed and replaced with MTT (3-(4,5-dimethylthiazol-2-yl)-2,5-diphenyltetrazolium bromide) solution (0.5 mg/mL in PBS) followed by incubation of cells for 4 h at 37° C. Next, 150 μL of DMSO was added to each well then absorbance readings (at 570 nm) were recorded using a microplate reader (TECANA-5082, Magellan, Austria).

### Glucose-stimulated insulin secretion (GSIS)

INS-1 cells (1 × 10^5^ cells/well) were seeded into wells of 96-well plates containing glucose (5 or 30 mM) followed by incubation for 48 h then incubation with 0, 50, 100, 200 μg/mL JDTL for 24 h. Thereafter, the cells were washed thoroughly with PBS followed by incubation in Krebs-Ringer bicarbonate HEPES buffer (KRBB, 4.8 mM KCl, 129 mM NaCl, 2.5 mM CaCl2, 1.2 mM KH2PO4, 1.2 mM MgSO4, 20 mM HEPES, 5 mM NaHCO3, and 0.5% bovine serum albumin (BSA), pH 7.4) for 1 h. The buffer was replaced with KRBB buffer containing 5 mmol/L glucose (LG) or 30 mmol/L glucose (HG) followed by incubation at 37° C for 1 h. Next, the supernatant was collected then the insulin concentration in the supernatant was determined using a Rat/Mouse Insulin Elisa Kit (Millipore, USA).

### Glucose uptake assay

HepG2 cells were seeded into wells of 24-well culture plates followed by incubation for 24 h. Next, cells were incubated with different drugs for 24 h then the medium was removed and the cells were washed once with PBS followed by incubation with 100 nM insulin for 30 min. After incubation, cells were incubated in 50 μM 2-NBDG for another 30 min then the supernatant was discarded and cells were washed twice with ice-cold PBS. Next, 2-NBDG fluorescence intensity was measured using a fluorescence microplate reader (Varioskan LUX, Thermo Fisher, USA) at an excitation wavelength of 485 nm and emission wavelength of 538 nm.

### Total RNA preparation and real-time PCR

Total RNA was extracted from INS-1 and HepG2 cells using TRIzol reagent (TIANGEN, Beijing, China) and reverse transcribed using the First Strand cDNA synthesis kit (TaKaRa, Dalian, China) according to manufacturer’s instructions. The RT-qPCR procedure was performed as follows: denaturation at 95° C for 15 min followed by 40 cycles that included denaturation at 95° C for 10 s, annealing at 60° C for 20 s, and elongation at 72° C for 30 s. The primers used to amplify PI3K, Akt, and GAPDH genes, were synthesized by Invitrogen (Thermo Fisher scientific, Inc.):

**Table:** 

PI3K	Forward GCTGTTGATAGACCACCGCTTCC
	Reverse TGCCCTGTTCCTCTGCCTTCC
Akt	Forward CAGGAGGAGGAGACGATGGACTTC
	Reverse CACACGGTGCTTGGGCTTGG
GAPDH	Forward TGGTGGACCTCATGGCCTAC
	Reverse CAGCAACTGAGGGCCTCTCT

The expression level of GAPDH was used as internal control.

### Western blot analysis

Western blot procedures were performed as previously described. First, INS-1 and HepG2 cells that received different treatments were harvested and lysed with radioimmunoprecipitation assay (RIPA) buffer (Beyotime Biotechnology, Jiangsu, China) supplemented with protease and phosphatase inhibitors (Roche, Mannheim, Germany). The following primary antibodies were used as probes: anti-IRS, anti-phosphor-Ser612-IRS-1, anti-PI3K, anti-phosphor-Thy458-PI3K, anti-Akt, anti-phosphor-Ser473-Akt, GIUT4, and anti-GAPDH, which were obtained from Cell Signaling Technology (Danvers, MA, USA). Quantification of relative changes in protein levels (arbitrary units expressed as percentage of the control protein level) was performed using Image J software.

### Statistical analysis

GraphPad Prism 6.0 software was used for statistical analysis of the data. Data are presented as mean ± SD from three different experiments. The results were considered statistically significant at *P* < 0.05.

### Availability of data and materials

The raw data supporting the conclusions of this article will be made available by the authors, without undue reservation, to any qualified researcher.

## Supplementary Material

Supplementary Table 1
