# Supplementary material for: Decoding the chemical composition and pharmacological mechanisms of Jiedu Tongluo Tiaogan Formula using high-performance liquid chromatography coupled with network pharmacology-based investigation
Source: Aging (Albany NY). 2021 Nov 5;13(21):24290–312. doi: 10.18632/aging.203679 (PMC8610129; doi:10.18632/aging.203679)
Supplement: Supplementary Table 1 [file aging-13-203679-s001.docx]

**Supplementary Table 1. Potential active ingredients of JDTL.**

| Mol ID | Compound | OB | DL | Medicine |
| --- | --- | --- | --- | --- |
| MOL002235 | EUPATIN | 50.8 | 0.41 | Dahuang |
| MOL002268 | rhein | 47.07 | 0.28 | Dahuang |
| MOL002281 | Toralactone | 46.46 | 0.24 | Dahuang |
| MOL002297 | Daucosterol_qt | 35.89 | 0.7 | Dahuang |
| MOL000471 | aloe-emodin | 83.38 | 0.24 | Dahuang |
| MOL000096 | (-)-catechin | 49.68 | 0.24 | Dahuang |
| MOL002251 | Mutatochrome | 48.64 | 0.61 | Dahuang |
| MOL002259 | Physciondiglucoside | 41.65 | 0.63 | Dahuang |
| MOL002260 | Procyanidin B-5,3'-O-gallate | 31.99 | 0.32 | Dahuang |
| MOL002276 | Sennoside E_qt | 50.69 | 0.61 | Dahuang |
| MOL002280 | Torachrysone-8-O-beta-D-(6'-oxayl)-glucoside | 43.02 | 0.74 | Dahuang |
| MOL002288 | Emodin-1-O-beta-D-glucopyranoside | 44.81 | 0.8 | Dahuang |
| MOL002293 | Sennoside D_qt | 61.06 | 0.61 | Dahuang |
| MOL002303 | palmidin A | 32.45 | 0.65 | Dahuang |
| MOL000358 | beta-sitosterol | 36.91 | 0.75 | Dahuang |
| MOL000554 | Gallic acid-3-O-(6'-O-galloyl)-glucoside | 30.25 | 0.67 | Dahuang |
| MOL004718 | α-spinasterol | 42.98 | 0.76 | Chaihu |
| MOL000449 | Stigmasterol | 43.83 | 0.76 | Chaihu |
| MOL004653 | (+)-Anomalin | 46.06 | 0.66 | Chaihu |
| MOL004598 | 3,5,6,7-tetramethoxy-2-(3,4,5-trimethoxyphenyl)chromone | 31.97 | 0.59 | Chaihu |
| MOL004609 | Areapillin | 48.96 | 0.41 | Chaihu |
| MOL001645 | Linoleyl acetate | 42.1 | 0.2 | Chaihu |
| MOL004624 | Longikaurin A | 47.72 | 0.53 | Chaihu |
| MOL004609 | Areapillin | 48.96 | 0.41 | Chaihu |
| MOL013187 | Cubebin | 57.13 | 0.64 | Chaihu |
| MOL000354 | isorhamnetin | 49.6 | 0.31 | Chaihu |
| MOL004628 | Octalupine | 47.82 | 0.28 | Chaihu |
| MOL004644 | Sainfuran | 79.91 | 0.23 | Chaihu |
| MOL001454 | berberine | 36.86 | 0.78 | Huanglian |
| MOL013352 | Obacunone | 43.29 | 0.77 | Huanglian |
| MOL002894 | berberrubine | 35.74 | 0.73 | Huanglian |
| MOL002897 | epiberberine | 43.09 | 0.78 | Huanglian |
| MOL002903 | (R)-Canadine | 55.37 | 0.77 | Huanglian |
| MOL002904 | Berlambine | 36.68 | 0.82 | Huanglian |
| MOL002907 | Corchoroside A_qt | 104.95 | 0.78 | Huanglian |
| MOL000622 | Magnograndiolide | 63.71 | 0.19 | Huanglian |
| MOL000762 | Palmidin A | 35.36 | 0.65 | Huanglian |
| MOL000785 | palmatine | 64.6 | 0.65 | Huanglian |
| MOL001458 | coptisine | 30.67 | 0.86 | Huanglian |
| MOL002668 | Worenine | 45.83 | 0.87 | Huanglian |
| MOL008647 | Moupinamide | 86.71 | 0.26 | Huanglian |
| MOL000211 | Mairin | 55.38 | 0.78 | Huangqi |
| MOL000239 | Jaranol | 50.83 | 0.29 | Huangqi |
| MOL000296 | hederagenin | 36.91 | 0.75 | Huangqi |
| MOL000033 | (3S,8S,9S,10R,13R,14S,17R)-10,13-dimethyl-17-[(2R,5S)-5-propan-2-yloctan-2-yl]-2,3,4,7,8,9,11,12,14,15,16,17-dodecahydro-1H-cyclopenta[a]phenanthren-3-ol | 36.23 | 0.78 | Huangqi |
| MOL000371 | 3,9-di-O-methylnissolin | 53.74 | 0.48 | Huangqi |
| MOL000374 | 5'-hydroxyiso-muronulatol-2',5'-di-O-glucoside | 41.72 | 0.69 | Huangqi |
| MOL000378 | 7-O-methylisomucronulatol | 74.69 | 0.3 | Huangqi |
| MOL000379 | 9,10-dimethoxypterocarpan-3-O-β-D-glucoside | 36.74 | 0.92 | Huangqi |
| MOL000380 | (6aR,11aR)-9,10-dimethoxy-6a,11a-dihydro-6H-benzofurano[3,2-c]chromen-3-ol | 64.26 | 0.42 | Huangqi |
| MOL000387 | Bifendate | 31.1 | 0.67 | Huangqi |
| MOL000392 | formononetin | 69.67 | 0.21 | Huangqi |
| MOL000398 | isoflavanone | 109.99 | 0.3 | Huangqi |
| MOL000417 | Calycosin | 47.75 | 0.24 | Huangqi |
| MOL000433 | FA | 68.96 | 0.71 | Huangqi |
| MOL000438 | (3R)-3-(2-hydroxy-3,4-dimethoxyphenyl)chroman-7-ol | 67.67 | 0.26 | Huangqi |
| MOL000439 | isomucronulatol-7,2'-di-O-glucosiole | 49.28 | 0.62 | Huangqi |
| MOL001601 | 1,2,5,6-tetrahydrotanshinone | 38.75 | 0.36 | Danshen |
| MOL001659 | Poriferasterol | 43.83 | 0.76 | Danshen |
| MOL001771 | poriferast-5-en-3beta-ol | 36.91 | 0.75 | Danshen |
| MOL001942 | isoimperatorin | 45.46 | 0.23 | Danshen |
| MOL002222 | sugiol | 36.11 | 0.28 | Danshen |
| MOL002651 | Dehydrotanshinone II A | 43.76 | 0.4 | Danshen |
| MOL002776 | Baicalin | 40.12 | 0.75 | Danshen,Chaihu |
| MOL000569 | digallate | 61.85 | 0.26 | Danshen |
| MOL000006 | luteolin | 36.16 | 0.25 | Danshen |
| MOL006824 | α-amyrin | 39.51 | 0.76 | Danshen |
| MOL007036 | 5,6-dihydroxy-7-isopropyl-1,1-dimethyl-2,3-dihydrophenanthren-4-one | 33.77 | 0.29 | Danshen |
| MOL007041 | 2-isopropyl-8-methylphenanthrene-3,4-dione | 40.86 | 0.23 | Danshen |
| MOL007045 | 3α-hydroxytanshinoneⅡa | 44.93 | 0.44 | Danshen |
| MOL007048 | (E)-3-[2-(3,4-dihydroxyphenyl)-7-hydroxy-benzofuran-4-yl]acrylic acid | 48.24 | 0.31 | Danshen |
| MOL007049 | 4-methylenemiltirone | 34.35 | 0.23 | Danshen |
| MOL007050 | 2-(4-hydroxy-3-methoxyphenyl)-5-(3-hydroxypropyl)-7-methoxy-3-benzofurancarboxaldehyde | 62.78 | 0.4 | Danshen |
| MOL007051 | 6-o-syringyl-8-o-acetyl shanzhiside methyl ester | 46.69 | 0.71 | Danshen |
| MOL007058 | formyltanshinone | 73.44 | 0.42 | Danshen |
| MOL007059 | 3-beta-Hydroxymethyllenetanshiquinone | 32.16 | 0.41 | Danshen |
| MOL007061 | Methylenetanshinquinone | 37.07 | 0.36 | Danshen |
| MOL007063 | przewalskin a | 37.11 | 0.65 | Danshen |
| MOL007064 | przewalskin b | 110.32 | 0.44 | Danshen |
| MOL007068 | Przewaquinone B | 62.24 | 0.41 | Danshen |
| MOL007069 | przewaquinone c | 55.74 | 0.4 | Danshen |
| MOL007070 | (6S,7R)-6,7-dihydroxy-1,6-dimethyl-8,9-dihydro-7H-naphtho[8,7-g]benzofuran-10,11-dione | 41.31 | 0.45 | Danshen |
| MOL007071 | przewaquinone f | 40.31 | 0.46 | Danshen |
| MOL007077 | sclareol | 43.67 | 0.21 | Danshen |
| MOL007079 | tanshinaldehyde | 52.47 | 0.45 | Danshen |
| MOL007081 | Danshenol B | 57.95 | 0.56 | Danshen |
| MOL007082 | Danshenol A | 56.97 | 0.52 | Danshen |
| MOL007085 | Salvilenone | 30.38 | 0.38 | Danshen |
| MOL007088 | cryptotanshinone | 52.34 | 0.4 | Danshen |
| MOL007093 | dan-shexinkum d | 38.88 | 0.55 | Danshen |
| MOL007094 | danshenspiroketallactone | 50.43 | 0.31 | Danshen |
| MOL007098 | deoxyneocryptotanshinone | 49.4 | 0.29 | Danshen |
| MOL007100 | dihydrotanshinlactone | 38.68 | 0.32 | Danshen |
| MOL007101 | dihydrotanshinoneⅠ | 45.04 | 0.36 | Danshen |
| MOL007105 | epidanshenspiroketallactone | 68.27 | 0.31 | Danshen |
| MOL007107 | C09092 | 36.07 | 0.25 | Danshen |
| MOL007108 | isocryptotanshi-none | 54.98 | 0.39 | Danshen |
| MOL007111 | Isotanshinone II | 49.92 | 0.4 | Danshen |
| MOL007115 | manool | 45.04 | 0.2 | Danshen |
| MOL007118 | microstegiol | 39.61 | 0.28 | Danshen |
| MOL007119 | miltionone Ⅰ | 49.68 | 0.32 | Danshen |
| MOL007120 | miltionone Ⅱ | 71.03 | 0.44 | Danshen |
| MOL007121 | miltipolone | 36.56 | 0.37 | Danshen |
| MOL007122 | Miltirone | 38.76 | 0.25 | Danshen |
| MOL007123 | miltirone Ⅱ | 44.95 | 0.24 | Danshen |
| MOL007124 | neocryptotanshinone ii | 39.46 | 0.23 | Danshen |
| MOL007125 | neocryptotanshinone | 52.49 | 0.32 | Danshen |
| MOL007127 | 1-methyl-8,9-dihydro-7H-naphtho[5,6-g]benzofuran-6,10,11-trione | 34.72 | 0.37 | Danshen |
| MOL007130 | prolithospermic acid | 64.37 | 0.31 | Danshen |
| MOL007132 | (2R)-3-(3,4-dihydroxyphenyl)-2-[(Z)-3-(3,4-dihydroxyphenyl)acryloyl]oxy-propionic acid | 109.38 | 0.35 | Danshen |
| MOL007140 | (Z)-3-[2-[(E)-2-(3,4-dihydroxyphenyl)vinyl]-3,4-dihydroxy-phenyl]acrylic acid | 88.54 | 0.26 | Danshen |
| MOL007141 | salvianolic acid g | 45.56 | 0.61 | Danshen |
| MOL007142 | salvianolic acid j | 43.38 | 0.72 | Danshen |
| MOL007143 | salvilenone Ⅰ | 32.43 | 0.23 | Danshen |
| MOL007145 | salviolone | 31.72 | 0.24 | Danshen |
| MOL007149 | NSC 122421 | 34.49 | 0.28 | Danshen |
| MOL007150 | (6S)-6-hydroxy-1-methyl-6-methylol-8,9-dihydro-7H-naphtho[8,7-g]benzofuran-10,11-quinone | 75.39 | 0.46 | Danshen |
| MOL007151 | Tanshindiol B | 42.67 | 0.45 | Danshen |
| MOL007152 | Przewaquinone E | 42.85 | 0.45 | Danshen |
| MOL007154 | tanshinone iia | 49.89 | 0.4 | Danshen |
| MOL007155 | (6S)-6-(hydroxymethyl)-1,6-dimethyl-8,9-dihydro-7H-naphtho[8,7-g]benzofuran-10,11-dione | 65.26 | 0.45 | Danshen |
| MOL007156 | tanshinone Ⅵ | 45.64 | 0.3 | Danshen |
| MOL000354 | isorhamnetin | 49.6 | 0.31 | Chaihu,Huangqi |
| MOL000422 | kaempferol | 41.88 | 0.24 | Chaihu,Huangqi,Zhenhua |
| MOL000098 | quercetin | 46.43 | 0.28 | Chaihu,Huanglian,Zhenhua,Huangqi |
| MOL000358 | beta-sitosterol | 36.91 | 0.75 | Dahuang,Zhenhau |
